# Allelic losses on chromosome 3p are accumulated in relation to morphological changes of lung adenocarcinoma

**DOI:** 10.1038/sj.bjc.6602005

**Published:** 2004-08-03

**Authors:** H Iijima, Y Tomizawa, K Dobashi, R Saito, T Nakajima, M Mori

**Affiliations:** 1 Department of Medicine and Molecular Science, Gunma University Graduate School of Medicine, 3-39-15 Showa-machi, Maebashi, Gunma 371-8511, Japan; 2 Department of Tumor Pathology, Gunma University Graduate School of Medicine, 3-39-15 Showa-machi, Maebashi, Gunma 371-8511, Japan

**Keywords:** loss of heterozygosity, differentiation, histological subtype, lung adenocarcinoma, chromosome 3p

## Abstract

We performed allelotyping analysis at nine regions on chromosome 3p using 56 microdissected samples from 23 primary lung adenocarcinomas to examine the process of progression within individual lung adenocarcinoma with various grades of differentiation. Identical allelic patterns among various grades of differentiation were found in eight cases. Accumulation of allelic losses from high to lower differentiated portions was found in seven cases and accumulation of allelic losses from low to higher differentiated portions was found in five cases. Various allelic patterns among various grades of differentiation were found in three cases. These results suggested that allelic losses on 3p play an important role in morphological changes of lung adenocarcinomas. We also investigated the relationship between allelic losses on 3p and histological subtypes of lung adenocarcinoma. The frequencies of allelic losses at 3p14.2 and telomeric region of 3p21.3 were higher in papillary type tumour (nine out of 14, 64% and 11 out of 15, 73%) than in bronchioloalveolar carcinoma-type tumour (one out of 8, 13%; *P*=0.031 and four out of 12, 33%; *P* = 0.057). These results indicated that allelic losses at 3p14.2 and telomeric region of 3p21.3 are related to pattern of the proliferation of lung adenocarcinoma.

Lung cancer is the leading cause of cancer death among men and women and has recently become one of the most common malignancies in the world. It is categorised into four major types including adenocarcinoma, squamous cell carcinoma, large cell carcinoma and small cell carcinoma (Travis *et al*, 1999). Among these histological types of lung cancers, the incidence of adenocarcinoma is the highest in Japan and is gradually increasing, as it is in the United States and other economically advanced countries (Wingo *et al*, 1999). One of the most characteristic features of lung adenocarcinoma is the high degree of morphological heterogeneity. Therefore, it has been very difficult to characterise the morphological and genetic progression patterns of lung adenocarcinoma as clearly as those of colon cancers (Fearon and Vogelstein, 1990). Recently, it was reported that sequential molecular abnormalities were involved in the multistep development of squamous cell carcinoma of the lung (Wistuba *et al*, 1999, 2000). Multistep carcinogenesis from atypical adenomatous hyperplasia (AAH) to bronchioloalveolar carcinoma (BAC) might occur in lung adenocarcinoma (Noguchi *et al*, 1995; Niho *et al*, 1999; Yamasaki *et al*, 2000), and accumulation of genetic alterations occurs in sequential from high to lower differentiated portions in individual lung adenocarcinoma (Yatabe *et al*, 2000). However, the roles of genetic alterations in the differentiation of lung adenocarcinoma have not been fully clarified. Adenocarcinomas of the lung are divided into several subtypes including the BAC and papillary (PAP) types (Travis *et al*, 1999). The BAC type grows by replacing of alveolar lining cells. The PAP type shows predominant papillary growth. Moreover, it has been reported that the PAP type was worse prognosis than BAC type (Yokose *et al*, 2000). However, the roles of genetic alterations in the subtypes of lung adenocarcinoma have not been clarified.

Loss of heterozygosity (LOH) and cytogenetic abnormalities of chromosome 3p sequences are critical events in the pathogenesis of human cancers, including lung cancer (Hung *et al*, 1995; Zabarovsky *et al*, 2002). Multiple 3p regions have been identified as showing frequent allelic losses in lung and other cancers (Hibi *et al*, 1992; Wistuba *et al*, 1997; Maitra *et al*, 2001). The critical regions on chromosome 3p in primary lung cancer have been reported to be 3p25, 3p21.3, 3p14.2 and 3p12-cen based on detailed allelotyping studies (Hibi *et al*, 1992, Wistuba *et al*, 2000). Furthermore, homozygous deletions have been found in lung cancer cell lines in the 3p21.3, 3p14.2 and 3p12 regions (Zabarovsky *et al*, 2002). Since these regions are discontinuous, there are probably several different tumour suppressor genes (TSGs) in the 3p region. Allelic loss of 3p21.3 is an early event in the pathogenesis of squamous cell lung cancer (Wistuba *et al*, 1999, 2000). However, the significance of allelic losses of these critical regions in the pathogenesis of lung adenocarcinoma has not been clarified.

In the present study, we performed a detailed allelotyping analysis at nine regions on chromosome 3p arm in 56 portions with various grades of differentiation from 23 lung adenocarcinoma patients using a microdissection technique to examine the process of progression within individual lung adenocarcinoma. We also investigated the relationship between allelic losses on chromosome 3p and histological subtypes of lung adenocarcinoma.

## MATERIALS AND METHODS

### Archival tumour specimens

In all, 23 primary lung adenocarcinomas were obtained from patients undergoing surgical resection between 1991 and 2001 at the Gunma University School of Medicine Hospital, Gunma, Japan and the National Nishigunma Hospital, Gunma, Japan. Histology slides from each case were reviewed, and the diagnosis of adenocarcinoma with mixed subtypes and the grade of differentiation were confirmed using the World Health Organization histological classification (Travis *et al*, 1999). All samples contained regions with various growth pattern and grades of differentiation. We defined that well-differentiated portions were composed of the BAC or PAP pattern, moderately differentiated portions were composed of the PAP or acinar pattern and poorly differentiated portions were composed of the solid pattern. According to this classification, 23 primary lung adenocarcinoma samples included 17 well-differentiated portions, 19 moderately differentiated portions and 20 poorly differentiated portions. The features of the histological subtypes in each portion were determined, excluding poorly differentiated portions. We defined that the BAC types were composed of a well-differentiated portion with the BAC pattern and moderately differentiated portions reminiscent of the BAC pattern. We defined that the PAP types were well and moderately differentiated papillary adenocarcinoma defined by architectural patterns and cytologic features. According to this classification, 36 well or moderately differentiated tumour portions were histologically classified as 17 BAC types and 19 PAP types. Serial 5-*μ*m sections were cut from archival, formalin-fixed, paraffin-embedded tissue. All slides were stained with haematoxylin and eosin, and one of the slides was coverslipped. The coverslipped slide was used as a guide to localise regions of interest for microdissection of the other slides.

### Archival specimen microdissection and DNA extraction

**Figure 1:**
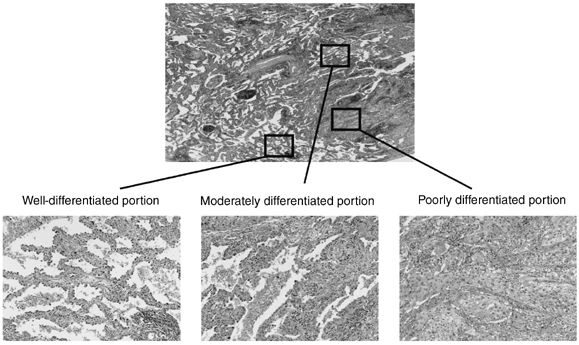
Representative example of lung adenocarcinoma used in laser microdissection. This case shows contiguous portions of well-, moderately and poorly differentiated tumours.

Microdissection from archival paraffin-embedded tissues was performed using a Leica laser microdissection system (Leica, Wetzlar, Germany) from multiple microslides of each sample (Figure 1

). DNA extraction was performed by transferring samples to a tube containing 45 *μ*l of proteinase K buffer (10 mM Tris-HCl pH=8.0, 1% Tween-20, 5 mg ml^−1^ proteinase K) and lysing cells at 50°C overnight, then incubating the lysate at 99°C for 10 min to inactivate the proteinase K. Dissected nonmalignant lung tissue from the same slides was used as a source of normal DNA from each case. After DNA extraction, 3 *μ*l of the proteinase K-digested samples containing DNA from at least 200 cells were used for each PCR reaction.

### Polymorphic DNA markers and PCR-LOH analysis

To evaluate LOH, we used primers flanking dinucleotide and multinucleotide microsatellite repeat polymorphisms spanning the entire chromosome 3p arm. The nine polymorphic markers used were distributed as follows: 3p12 (*D3S1274*), 3p14.2 (*D3S1234*), 3p21.2 – 21.3 (*D3S1573*), centromeric region of 3p21.3 (3p21.3C) (*D3S4622*, *D3S4597*), telomeric region of 3p21.3 (3p21.3T) (*D3S1478*), 3p22 – 24 (*D3S1612*, *D3S2432*) and 3p25 (*D3S1597*). Primer sequences were obtained from the Genome Database (http://www.gdb.org/gdb/) for all these markers. Two-round PCR methods were used. The first PCR amplifications were carried out in a 20 *μ*l reaction mixture containing 1.5 mM MgCl_2_, 0.5 *μ*M of each primer, 0.2 mM each deoxynucleotide triphosphate, and 1 U of Hotstar*Taq* DNA polymerase (QIAGEN, Valencia, CA, USA). The second PCR amplifications were carried out in a 20 *μ*l reaction mixture containing 0.5 *μ*M of each primer, which were used in the first PCR reaction, 0.1 *μ*l of [*α*-32P]dCTP (3000 Ci mmol^−1^, 10 mCi ml^−1^), and 3 *μ*l of the first PCR product. For both PCR reactions, after initial denaturation at 95°C for 15 min, 40 cycles each consisting of denaturation at 94°C for 30 s, annealing at 50–57°C for 30 s, strand elongation at 72°C for 1 min were carried out, followed by a final elongation 72°C for 5 min. PCR products were diluted five-fold with denaturing buffer containing 95% formamide, 20 mM EDTA and dye, and denatured at 90°C for 3 min. A volume of 2 *μ*l of the denatured sample was electrophoresed on a 5% denaturing polyacrylamide gel at 1500 V constant voltage for 2 h at room temperature. Gels were dried and exposed to X-ray film at room temperature. LOH was scored by visual detection of the complete absence of one allele in autoradiographs.

### Homozygous deletion analysis

We found homozygous deletion at 3p25 (*D3S1597*) in two poorly differentiated tumour samples by PCR-LOH analysis. To confirm that the homozygous deletion centred on *D3S1597*, we performed multiplex PCR analysis involving the amplification of two different sets of primer pairs in the same reaction mixture. The *D3S1234* primer pair was used as a control to test for homozygous deletion centred on *D3S1597*. Two primer sets (*D3S1597* and *D3S1234*) were used in the same reaction to ensure the amplification of the DNA and normalise the amount of products from the primary lung cancer DNA relative to that of the non-small cell lung cancer cell line H1666 for both the test and control primers. The PCR reaction was performed as described above for LOH assays. After amplification, PCR products were separated by electrophoresis on a 2% agarose gel and visualised by ethidium bromide staining.

### Statistical analysis

Fisher's exact test was used to examine the association of two categorical variables. A *P*-value of less than 0.05 was considered to be statistically significant. Statistical analysis was performed using StatView J-4.5 for Macintosh.

## RESULTS

### Allelic losses of chromosome 3p in morphological changes of lung adenocarcinoma with various grades of differentiation in individuals

**Figure 2:**
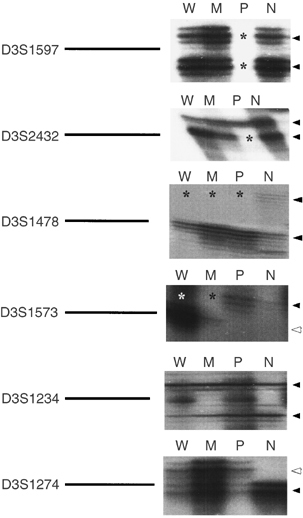
Representative autoradiographs of microsatellite analyses for LOH using multiple 3p markers in the microdissected primary lung adenocarcinoma and normal epithelium from a cancer patient (No. 477). The markers are indicated on the left of the autoradiograph panel. Black arrowheads indicate main allele bands and white arrowheads indicate microsatellite instability bands. Asterisks indicate allelic loss. Microsatellite instabilities were seen at *D3S1573* and *D3S1274*. W=well-differentiated portion; M=moderately differentiated portion; P=poorly differentiated portion; N=histologically normal epithelium.

LOH of chromosome 3p was examined in 56 portions with various grades of differentiation from 23 lung adenocarcinomas by PCR amplification using nine microsatellite polymorphic markers distributed across chromosome 3p. Representative results of LOH analysis are shown in Figure 2

**Figure 3:**
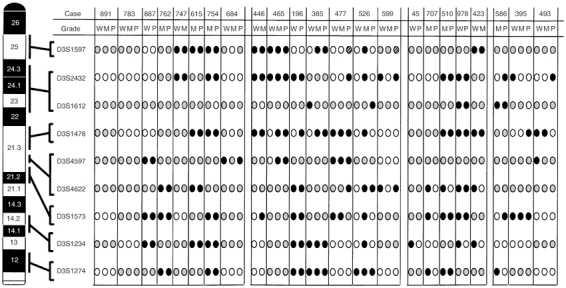
Left: diagram of the short arm of chromosome 3 (3p) showing the nine microsatellite markers used in the allelotyping analysis. Their order and approximate locations are derived from the Genome Database. Right: summary of all 3p allelotyping results with nine microsatellite markers in lung adenocarcinoma. Solid circles, LOH; open circles, retention of heterozygosity; grey circle, noninformative; hatched circles, homozygous deletion. For each individual tumour analysed, the case number and corresponding grade of differentiation are indicated above the lanes. W=well-differentiated portion; M=moderately differentiated portion; P=poorly differentiated portion.

. We first examined the relationship between the patterns of 3p LOH and various grades of differentiation within individual tumours. Allelic patterns of 3p in all samples are shown in Figure 3

**Figure 4:**
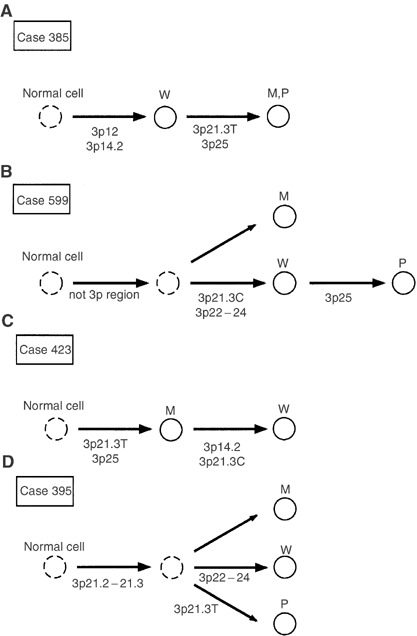
Representative diagrams of the progression among various grades of differentiation in lung adenocarcinoma. The loci of allelic losses are shown under the arrows. W=well-differentiated portion; M=moderately differentiated portion; P=poorly differentiated portion. 3p21.3T=telomeric region of 3p21.3. 3p21.3C=centromeric region of 3p21.3.

. Identical allelic patterns among well-, moderately and poorly differentiated portions in individuals were found in eight cases (cases 891, 783, 887, 762, 747, 615, 754 and 684). Accumulation of LOH from well- or moderately to moderately or poorly differentiated portions were found in cases 446, 465, 196, 385 and 477. For example case 385, though LOHs at *D3S1234* and *D3S1274* were found in every differentiated portion, LOHs at *D3S1597* and *D3S1478* were found in moderately and poorly differentiated portions but not in well-differentiated portion (Figure 3). This implied that moderately and poorly differentiated portions of this tumour were expanded from well-differentiated portions with accumulation of LOHs at 3p25 and 3p21.3T (Figure 4A

). In cases 526 and 599, whereas the allelic pattern of the moderately differentiated portion differed from those of the well- and poorly differentiated portions, LOH or homozygous deletion was accumulated from the well-differentiated portion to poorly differentiated portion (Figure 3). For example case 599, there was no LOH in moderately differentiated portions. Although LOHs at *D3S2432* and *D3S4622* were found in well- and poorly differentiated portions, homozygous deletion at *D3S1597* was found only in poorly differentiated portion. It is possible that moderately differentiated portion was progressed different from well- and poorly differentiated portions, and poorly differentiated portion was expanded from well-differentiated portion with accumulation of allelic loss at 3p25 (Figure 4B). On the other hand, accumulation of LOH from the poorly or moderately to moderately or well-differentiated portions were found in cases 45, 707, 510, 978 and 423 (Figure 3). For example case 423, though LOHs at *D3S1597* and *D3S1478* were found in well- and moderately differentiated portions, LOHs at *D3S4622* and *D3S1234* were found only in well-differentiated portion. This implied that well-differentiated portion of this tumour was expanded from moderately differentiated portions with accumulation of LOHs at 3p21.3C and 3p14.2 (Figure 4C). Various LOH patterns among various grades of differentiation in individuals were found in cases 586, 395 and 493 (Figure 3). For example case 395, though the allelic patterns at *D3S1573* and *D3S1234* was identical among well-, moderately and poorly differentiated portions, LOH at *D3S2432* was shown only in well-differentiated portion and LOH at *D3S1478* was shown only in poorly differentiated portion. It is possible that common precursor region of the tumours occurred with LOH at 3p21.2–21.3, and well- and poorly differentiated portions were expanded from the common precursor region with accumulation of LOHs at 3p22–24 and 3p21.3T (Figure 4D).

### Allelic losses of chromosome 3p in lung adenocarcinomas with various grades of differentiation

We next examined the correlation between LOHs at each locus on chromosome 3p and various grades of differentiation to find the regions relating differentiation of lung adenocarcinoma. Allelic losses involving one or more 3p regions were detected in 21 out of 23 cases (91%) (Figure 3). The frequencies of LOH at one or more 3p loci were 15 out of 17 (88%) in well-differentiated (W) portions, 15 out of 19 (79%) in moderately differentiated (M) portions, and 16 out of 20 (80%) in poorly differentiated (P) portions (Table 1

**Table 1:** Loss of heterozygosity at 9 chromosome 3p polymorphic markers in 56 variously differentiated portions of lung adenocarcinomas

		**LOH(%)**			
**Marker**	**Region**	**Total**	**W^a^**	**M^a^**	**P^a^**
***D3S1597***	**3p25**	**18/28(64.2)**	**4/9(44.4)**	**8/10(80.0)**	**6/9(66.7)**
*D3S2432*	3p22–24	22/47(46.8)	7/15(46.7)	6/15(40.0)	9/17(52.9)
*D3S1612*	3p22–24	6/8(75.0)	2/2(100)	1/1(100)	3/5(60.0)
***D3S1478***	**3p21.3**	**24/41(58.5)**	**5/13(38.4)**	**10/14(71.4)**	**9/14(64.2)**
*D3S4597*	3p21.3	10/14(71.4)	4/6(66.6)	2/3(66.6)	4/5(80.0)
*D3S4622*	3p21.3	16/21(76.2)	4/5(80.0)	5/7(71.4)	7/9(77.8)
*D3S1573*	3p21.2–21.3	21/34(61.8)	5/10(50.0)	8/12(66.7)	8/12(66.7)
*D3S1234*	3p14.2	15/35(42.9)	6/11(54.5)	4/11(36.4)	5/13(38.5)
*D3S1274*	3p12	16/35(45.7)	3/9(33.3)	7/13(53.8)	6/13(46.2)
	Any region	46/56(82.1)	15/17(88.2)	15/19(78.9)	16/20(80.0)

a W=well-differentiated portion; M=moderately differentiated portion; P=poorly differentiated portion.

). There were no differences of the frequency of LOH in any differentiation state. The frequencies of LOH at 3p25 (*D3S1597*) and 3p21.3T (*D3S1478*) loci in W (four out of nine, 44% and five out of 13, 38%) portions were lower than those in M (eight out of 10, 80% and 10 out of 14, 71%) and P (six out of nine, 67% and nine out of 14, 64%) portions (Table 1). However, the differences did not reach the statistically significance (*P*=0.2096 and 0.0978). Notably, homozygous deletions were found at 3p25 (*D3S1597*) only in two poorly differentiated portions (cases 477 and 599) (Figure 3). These homozygous deletions were confirmed by at least three independent repetitions of PCR and gel electrophoresis. DNA integrity was shown by successful amplification of another microsatellite marker (*D3S1234*) by multiplex PCR (data not shown).

### Allelic losses of chromosome 3p in the subtypes of lung adenocarcinomas

We then analysed the relationship between 3p LOH and histological subtypes of lung adenocarcinoma. The poorly differentiated tumours were excluded from this analysis. The frequencies of LOH at one or more 3p loci were 18 out of 19 (95%) in PAP-type tumours and 12 out of 17 (71%) in BAC-type tumours (Table 2

**Table 2:** Frequencies of loss of heterozygosity according to histological subtype

		**LOH (%)**	
**Marker**	**Region**	**PAP^a^ type**	**BAC^a^ type**
*D3S1597*	3p25	8/12(66.7)	4/7(57.1)
*D3S2432*	3p22–24	6/13(46.2)	7/17(41.2)
*D3S1612*	3p22–24	2/2(100)	1/1(100)
***D3S1478***	**3p21.3**	**11/15(73.3)**	**4/12(33.3)**
*D3S4597*	3p21.3	2/2(100)	4/7(57.1)
*D3S4622*	3p21.3	7/9(77.8)	2/3(66.7)
*D3S1573*	3p21.2–21.3	7/11(63.6)	6/11(54.5)
***D3S1234***	**3p14.2**	**9/14(64.3)**	**1/8(12.5)**
*D3S1274*	3p12	8/14(57.1)	2/8(25.0)
	Any region	18/19(94.7)	12/17(70.6)

Poorly differentiated tumours were excluded from this analysis.

a PAP=papillary; BAC=bronchioloalveolar carcinoma.

). The frequency of LOH at 3p14.2 (*D3S1234*) in PAP-type tumours (nine out of 14, 64%) was significantly higher than that in BAC-type tumours (one out of eight, 13%) (*P*=0.031) (Table 2). The frequency of LOH at 3p21.3T (*D3S1478*) in PAP-type tumours (11 out of 15, 73%) was also higher than that in BAC-type tumours (four out of 12, 33%) (*P*=0.0574). There were no differences of the frequency of LOH at other loci between PAP-type tumours and BAC-type tumours.

## DISCUSSION

Although the carcinogenesis and progression of lung adenocarcinoma has been reported to be a multistage process (Yamasaki *et al*, 2000; Yatabe *et al*, 2000; Aoyagi *et al*, 2001), the pathogenesis of lung adenocarcinoma has not been fully clarified. Therefore, we examined the pattern of 3p LOH among several differentiated portions within individuals. Eight cases showed identical allelic patterns in various grades of differentiation. It has been reported that several TSGs on other chromosomes are related to lung carcinogenesis and tumour development (Zabarovsky *et al*, 2002). Therefore, in cases showing genetic homogeneity despite great morphological divergence, genetic alterations might be present at loci other than those examined in the present study. Seven cases showed the accumulation of LOH from high to lower differentiated portions. It has been reported that accumulation of LOH occurred from high to lower differentiated portion (Yamasaki *et al*, 2000; Yatabe *et al*, 2000; Aoyagi *et al*, 2001). These results suggested that well-differentiated tumours progressed to more poorly differentiated tumours with accumulation of genetic alterations. On the other hand, five cases showed the accumulation of LOH from low to higher differentiated portions. It is possible that some poorly or moderately differentiated tumours progress to moderately or well-differentiated tumour. Interestingly, we found three cases showing various allelic patterns in tumour portions with various grades of differentiation and two cases showing different allelic patterns between moderately and well- or poorly differentiated portions. Although the progression of the grade of differentiation did not appear as a sequential process, identical allelic patterns were found in several markers. Therefore, it is possible that the progression of the grade of differentiation is not sequential, but each differentiated portion is expanded from the common precursor region in these cases. Another possibility is that other loci which were not analysed in the present study are more important in morphological changes of lung adenocarcinoma.

We demonstrated here that allelic losses at 3p25 and 3p21.3T tended to be more frequent in poorly or moderately differentiated portions than in well-differentiated portions of lung adenocarcinoma. Furthermore, homozygous deletions were found only in poorly differentiated portion. It has been reported that LOH on chromosome 3p, 9p and 17p and mutations of the *p53* gene were more frequent in more poorly differentiated portions of individual lung adenocarcinoma (Yamasaki *et al*, 2000). Based on macrodissection analysis, LOH on 3p in poorly differentiated or undifferentiated tumours was more frequent than that in well-differentiated tumours in the lung adenocarcinoma (Yokoyama *et al*, 1992). These results, including the present study, suggested that allelic loss on chromosome 3p, especially 3p25 and 3p21.3T is related to the differentiation of lung adenocarcinoma. On the other hand, Yatabe *et al* (2000) reported that LOH on 3p and 17p were concordant despite morphological diversity in five lung adenocarcinoma cases. The discrepancy between the present study and that previous study might be due to the difference of sample size. In fact, eight cases in the present study showed identical allelic patterns among variously differentiated portions in individuals.

Lung adenocarcinomas are divided into several subtypes including the BAC and PAP types (Travis *et al*, 1999). Recently, it was reported that the frequency of the *p53* mutations in BAC was lower than that in other subtypes, including PAP-type tumours (Koga *et al*, 2001). The frequency of LOH at 3p14.2 in localised BAC was lower than that in BAC with fibroblastic proliferation (Aoyagi *et al*, 2001). We demonstrated that allelic losses at the 3p14.2 and 3p21.3T regions were more frequent in PAP type than in BAC type lung adenocarcinoma. These results indicate that LOHs at 3p14.2 and 3p21.3T are related to pattern of the proliferation of lung adenocarcinoma.

In summary, we found that the morphological change of lung adenocarcinoma with various grades of differentiation in individuals was related to accumulation of allelic losses of chromosome 3p. Although we could not find out the critical regions which regulate differentiation of lung adenocarcinoma, deletions at 3p25 and 3p21.3T tended to be more frequent in tumours with more poorly differentiated portions. Especially, homozygous deletions were found at 3p25 in two poorly differentiated portions. Further analyses with large number of cases will be needed to find out the critical regions relating to differentiation of lung adenocarcinoma. We also found that deletions at 3p14.2 and 3p21.3T were more frequent in PAP-type tumours than in BAC-type tumour. This suggested that deletions at 3p14.2 and 3p21.3T play an important role in the pattern of the proliferation of lung adenocarcinoma.

In the future, our finding in the present study may be helpful for distinguishing metastases from second primary tumours in multiple synchronous lung adenocarcinoma by examining the genetic homogeneity within individual tumour, and be helpful for the diagnosis of histopathological classification of lung adenocarcinoma on tiny samples such as bronchial biopsies and fine-needle aspiration biopsies by examining the LOH pattern of 3p especially 3p25, 3p21.3T, 3p14.2. Additional molecular analyses with large number cases are necessary to further characterise the clinicopathological nature including prognosis of lung adenocarcinomas.
